# Knowledge, attitudes, and practices towards COVID-19 among undergraduates during emergency remote learning

**DOI:** 10.1007/s44155-022-00017-x

**Published:** 2022-08-01

**Authors:** Chin Xuan Tan, Shu Chyi Wong, Seok Shin Tan, Seok Tyug Tan

**Affiliations:** 1 Department of Allied Health Sciences, Faculty of Science, Universiti Tunku Abdul Rahman, Jalan Universiti Bandar Barat 31900, Kampar Perak, Malaysia; 2 Jeffrey Cheah School of Medicine and Health Sciences, Monash University Malaysia, 47500 Bandar Sunway, Selangor Malaysia; 3 Department of Healthcare Professional, Faculty of Health and Life Sciences, Management and Science University, University Drive, Off Persiaran Olahraga, Seksyen 13, 40100 Shah Alam, Selangor Malaysia

**Keywords:** COVID-19, Emergency remote learning, Knowledge, Attitude, Practice

## Abstract

**Background:**

The coronavirus disease 2019 (COVID-19) pandemic paralyzes the education sector. To minimize the interruption of teaching and learning, most universities in Malaysia shifted to virtual mode during this unprecedented period of the pandemic. With an ever-increasing number of Malaysians fully vaccinated against COVID-19, the education system is expected to switch back to face-to-face mode this year. It is crucial to assess the knowledge, attitudes, and practices (KAP) of COVID-19 among emergency remote learning undergraduates before reverting to physical teaching and learning. Hence, a study was conducted with this aim in mind.

**Methods:**

A total of 299 Malaysian undergraduates were recruited through a snowball sampling approach. The online questionnaire encompassed three main segments: informed consent, sociodemographic information, and KAP questions on COVID-19.

**Results:**

The mean scores for knowledge, attitude, and practice were 4.05/6, 11.14/12, and 5.07/7, respectively. The results of the present study showed that year 1 respondents had significantly higher levels (*p* < 0.05) of KAP scores than year 4 respondents. In addition, the attitude score of science majors respondents was significantly greater (*p* < 0.05) than those of nonscience majors. The KAP scores showed no significant difference among groups with different sexes, ethnicities, and COVID-19 histories. Partial correlation analysis revealed that the overall knowledge score was positively correlated with attitude (r = 0.193, p = 0.001) and practice (r = 0.343, p < 0.001) scores whereas the total attitude score was positively correlated with the total practice score (r = 0.149, p = 0.010).

**Conclusion:**

Our current results suggest that COVID-19 workshops, seminars, or training programs for year 4 students could be conducted to enhance their KAP levels.

## Introduction

Coronavirus disease 2019 (COVID-19) is a highly infectious disease caused by the SARS-CoV-2 virus. The disease has spread and mutated rapidly since the first outbreak in late December 2019 in Wuhan, China. The rapid spread of COVID-19 globally is attributable to its long incubation period, high transmission rate, and asymptomatic status among carriers [1]. The virus is mainly transmitted between people through contact routes and respiratory droplets [2].

The first positive COVID-19 case in Malaysia was confirmed on 25 January 2020 [3]. Since then, several COVID-19 outbreaks have been reported in various regions of Malaysia. To curb the spread of this disease, the Malaysian government implemented different phases of lockdown in stages over the past year [4]. This causes unprecedented disruption to all sectors. In the education sector, students were instructed to stay at home, and the process of teaching and learning switched to virtual mode [5].

Emergency remote learning (ERL) refers to a sudden change from face-to-face learning to online learning in the event of a crisis [6]. When the crisis subsides, the education system returns to its original setting [7]. Contrasting ERL with distance learning, it is specifically designed and planned for online platforms. ERL was implemented by most institutions during the outbreak of COVID-19 for continuity of the educational processes. With an ever-increasing number of Malaysians fully vaccinated against COVID-19, the education system is expected to switch back to face-to-face mode this year.

Starting 1 May 2022, a set of relaxed COVID-19 standard operating procedures is being implemented across Malaysia. Optional facemask wearing outdoors and teaching in class, physical distancing abolishment, *MySejahtera* (a mobile app for COVID-19 contact tracing) check-ins abolishment [8], among others. People may catch COVID-19 even though they have been fully vaccinated [9]. Compared to other demographic groups, undergraduates are very active in activities such as part-time work, volunteering, sports clubs, and societies [10], which pose a high risk of contracting COVID-19.

Knowledge, attitude, and practice (KAP) surveys are useful tools to elicit what is known, believed, and done in the context of the topic of interest [11]. In the public health setting, data obtained from KAP surveys are often used to provide information for resource allocation in the planning and implementation of interventions [11]. Understanding the KAP of COVID-19 among undergraduates is crucial before teaching and learning are reverted to the physical mode. This is to prevent the spread of this contagious disease in universities. Hence, this study aims to assess the KAP related to COVID-19 among undergraduates during emergency remote learning.

## Methodology

### Study design and respondents

This cross-sectional study was carried out using a snowball sampling technique in October 2021. The self-administered questionnaire was hosted on Google Forms, and the generated link was circulated to platforms such as Microsoft Teams, Facebook, Instagram, and WhatsApp. The minimum sample size, 264, was calculated using the Cochran formula with a prevalence rate of 22% [12], a margin of error of 5%, and a 95% confidence interval. A total of 299 Malaysian undergraduates were recruited after excluding those students who were in the foundation, diploma, and postgraduate studies. All respondents were required to log into their email accounts to participate in this survey. This is to prevent duplicate responses. To ensure the anonymity of respondents, the current study did not record any personal information. Ethical approval from the Universiti Tunku Abdul Rahman Scientific and Ethical Review Committee (protocol number: U/SERC/181/2021) was obtained.

### Survey instrument

The English version of the questionnaire encompassed three main segments: informed consent, sociodemographic information, and KAP questions on COVID-19. On the first page of the Google Form, the objectives, study background, eligible respondents’ criteria, declaration of confidentiality and anonymity, and statements describing the willingness to participate as voluntary were made available. Respondents provided their consent by selecting ‘agree’ before participating in this study. Information on sex, ethnicity, year of study, field of study, and COVID-19 history were collected in the sociodemographic segment. Meanwhile, questions to assess the KAP towards COVID-19 were adapted from Ferdous et al. [13]. The knowledge section comprised 6 items with the responses of yes, don’t know, and no. Each correct answer was coded 1, whereas an incorrect or uncertain response was coded 0. The total knowledge score ranged from 0 to 6. Meanwhile, the attitude section comprised 6 items rated on a 3-point Likert scale (agree, undecided, and disagree). The total attitude score ranged from 0 to 12, with 0 points for ‘disagree’, 1 point for ‘undecided’, and 2 points for ‘agree’ for each response in this section. Last, the practice section comprised 7 items with the responses of yes, sometimes, and no. Each correct answer was coded 1, whereas an incorrect response was coded 0. The total practice score ranged from 0 to 7.

### Statistical analysis

The data analysis was conducted using Microsoft Excel 2016 (Microsoft Corp., Redmond, WA, USA) and IBM Statistical Package for the Social Sciences (SPSS) software version 26 (IBM SPSS Statistics, Inc., Chicago, IL, USA). All data were sorted and coded in Microsoft Excel before importing into SPSS software. All quantitative variables were checked for normality and the variables with skewness in the range of ± 2 were considered normally distributed [14]. Descriptive data for categorical variables were reported as frequencies and percentages, whereas data for continuous variables were reported as the means and standard deviations (SD). The differences in the knowledge, attitude, and practice scores with the sociodemographic variables were examined using either an independent sample t test or a one-way analysis of variance followed by Tukey’s post-hoc test. Demographics are potential confounder variables [15]. After controlling for factors that were statistically significant in the bivariate analysis, a partial correlation was used to understand the relationship between KAP scores. The level of significance was set at *p* < 0.05.

## Results

### Sociodemographic characteristics

The sociodemographic characteristics of the respondents are summarized in Table 1. There were a total of 299 respondents (44.8% for males and 55.5% for females) recruited in this study. The majority of the respondents were Chinese (61.5%), year 3 (38.8%), science majors (51.8%), and without a COVID-19 history (82.9%).

**Table 1:** Characteristics of the respondents

Characteristic	n (%)
Sex	
Male	134 (44.8)
Female	165 (55.2)
Ethnicity	
Chinese	184 (61.5)
Malay	52 (17.4)
India	54 (18.1)
Other	9 (3.0)
Field of study	
Science	155 (51.8)
Nonscience	144 (48.2)
Year of study	
Year 1	77 (25.8)
Year 2	68 (22.7)
Year 3	116 (38.8)
Year 4	38 (12.7)
COVID-19 history	
Yes	51 (17.1)
No	248 (82.9)

### Knowledge, attitudes, and practices towards COVID-19

The responses to the KAP questions are summarized in Table 2. More than 60% of the respondents agreed that COVID-19 was a dangerous disease, affected only human beings, and could be transmitted from human beings to animals and vice versa. Meanwhile, 58.2% of the respondents believed that COVID-19 can be transmitted in animal products, whereas 54.8% of the respondents viewed that COVID-19 can be transmitted in well-cooked products.

**Table 2:** COVID-19 knowledge, attitudes, and practices

Knowledge component		Response, n (%)		
		Yes	Don’t know	No
K1	Is COVID-19 a dangerous disease?	278 (92.9)	13 (4.3)	8 (2.6)
K2	Does COVID-19 affect only humans?	201 (67.2)	29 (9.7)	69 (23.1)
K3	Does COVID-19 transmit from humans to animals?	190 (63.5)	63 (21.1)	46 (15.4)
K4	Does COVID-19 transmit from animals to humans?	205 (68.6)	54 (18.1)	40 (13.4)
K5	Is it transmitted by animal products (e.g., milk, meat)?	174 (58.2)	64 (21.4)	61 (20.4)
K6	Is COVID-19 transmitted in well-cooked products?	164 (54.8)	44 (14.8)	91 (30.4)

Attitude component		Response, n (%)		
		Agree	Undecided	Disagree
A1	Do you think that it is crucial to report a suspected case to health authorities?	281 (94.0)	13 (4.3)	5 (1.7)
A2	Do you think that it is important to use a face mask in crowded place?	275 (92.0)	21 (7.0)	3 (1.0)
A3	Do you think that it is important to wash hands and face after coming outsides?	275 (92.0)	23 (7.7)	1 (0.3)
A4	Do you think that COVID-19 is a preventable disease?	247 (82.6)	43 (14.4)	9 (3.0)
A5	Do you think that COVID-19 can be treated at home?	222 (74.2)	61 (20.4)	16 (5.4)
A6	Do you think that health education can play an important role in COVID-19 prevention?	272 (91.0)	27 (9.0)	0 (0)

Practice component		Response, n (%)		
		Yes	Sometimes	No
P1	Do you use tissues or handkerchiefs during coughing/sneezing?	217 (72.6)	60 (20.1)	22 (7.4)
P2	Do you wash hands frequently using water and soaps?	236 (78.9)	42 (14.0)	21 (7.0)
P3	Do you avoid touching face and eyes?	208 (69.6)	66 (22.1)	25 (8.4)
P4	Do you maintain social distance (or home quarantine)?	236 (78.9)	46 (15.4)	17 (5.7)
P5	Do you eat healthy food during the COVID-19 outbreak?	198 (66.2)	71 (23.7)	30 (10.0)
P6	Do you maintain a healthy lifestyle during the COVID-19 outbreak?	192 (64.2)	81 (27.1)	26 (8.7)
P7	Do you obey all government rules related to the COVID-19?	228 (76.3)	51 (17.1)	20 (6.7)

In the attitude components, more than 90% of the respondents answered that the suspected COVID-19 should be reported to health authorities, face masks should be worn when in a crowded place, hands and face should be washed after coming from outside, and COVID-19 cases can be reduced through health education. Meanwhile, 82.6% of the respondents believed that COVID-19 was a preventable disease, and 74.2% of the respondents viewed that COVID-19 can be treated at home.

Regarding practices, most of the respondents used tissues or handkerchiefs during coughing/sneezing (72.6%), washed hands frequently using water and soaps (78.9%), avoided touching the face and eyes (69.6%), maintained social distance or home quarantine (78.9%), ate healthy food (66.2%), maintained a healthy lifestyle (64.2%), and followed the government rules concerning COVID-19 (76.3%).

### Comparison of knowledge, attitude, and practice scores between different groups

Table 3 shows the differences in KAP scores according to the sociodemographic parameters. The overall scores for the knowledge, practice, and attitude components were 4.05 ± 1.90, 11.14 ± 1.29, and 5.07 ± 2.27, respectively. It was found that the attitude score of science majors respondents was significantly greater (*p* < 0.05) than those of nonscience majors. The KAP scores of year 1 respondents were significantly greater (*p* < 0.05) than those of year 4 respondents. The current study indicated no difference in the KAP scores between year 2 and year 3 respondents. However, the attitude and practice scores of year 4 respondents were significantly lower (*p* < 0.05) than those of year 2 and year 3 respondents. Other parameters, such as sex, ethnicity, and COVID-19 history exhibited no significant difference in the KAP scores.

**Table 3:** Bivariate analysis of the factors associated with the knowledge, attitudes, and practices towards COVID-19

**Parameter**	Knowledge score		Attitude score		Practice score	
	Mean (SD)	t/F (*p* value)	Mean (SD)	t/F (*p* value)	Mean (SD)	t/F (*p* value)
Sex						
Male	4.17 ± 1.95	− 0.970 (0.333)	11.15 ± 1.27	− 0.065 (0.948)	4.97 ± 2.33	0.663 (0.508)
Female	3.96 ± 1.85		11.14 ± 1.32		5.15 ± 2.22	
Ethnic ity^†^						
Chinese	3.93 ± 1.86^a^	1.619 (0.185)	11.23 ± 1.06^a^	1.130 (0.337)	5.17 ± 2.16^a^	0.450 (0.717)
Malay	4.54 ± 1.89^a^		11.13 ± 1.66^a^		4.94 ± 2.48^a^	
India	4.09 ± 1.95^a^		10.91 ± 1.56^a^		4.94 ± 2.41^a^	
Other	3.56 ± 2.24^a^		10.78 ± 1.64^a^		4.44 ± 2.60^a^	
Field of study						
Science	4.13 ± 1.89	0.652 (0.515)	11.36 ± 1.04	3.006 (0.003)	4.89 ± 2.38	− 1.397 (0.163)
Nonscience	3.98 ± 1.90		10.91 ± 1.50		5.26 ± 2.14	
Year of study^†^						
Year 1	4.71 ± 1.85^a^	4.726 (0.003)	11.74 ± 0.59^a^	11.889 (0.000)	5.64 ± 2.22^a^	3.552 (0.015)
Year 2	3.72 ± 2.16^b^		11.16 ± 1.32^b^		4.75 ± 2.37^a^	
Year 3	3.95 ± 1.70^b^		11.00 ± 1.37^b^		5.12 ± 2.22^a^	
Year 4	3.63 ± 1.79^b^		10.34 ± 1.55^c^		4.32 ± 2.15^b^	
COVID-19 history						
Yes	4.45 ± 1.77	− 1.647 (0.101)	10.98 ± 1.36	0.986 (0.325)	5.20 ± 2.25	− 0.446 (0.656)
No	3.97 ± 1.92		11.18 ± 1.28		5.04 ± 2.28	

^†^Means within a column with different superscripts are significantly different at *p* < 0.05 from each other, based on Tukey’s post-hoc analysis

### Correlation between knowledge, attitude, and practice scores

The results of zero-order and partial correlations between KAP scores are shown in Table 4. Strengths of correlations are interpreted using the following criteria: > 0.90: very strong correlation, 0.70–0.89: strong correlation, 0.40 – 0.69: moderate correlation, and < 0.39: weak correlation [16]. Sociodemographic variables that were statistically significant in the bivariate analysis (Table 3) were the controlled variables in partial correlation. The overall knowledge score exhibited weak, positive correlations with attitude (r = 0.193, p = 0.001) and practice (r = 0.343, p < 0.001) scores after controlling for field of study and year of study. Meanwhile, the total attitude score demonstrated a weak, positive correlation with the total practice score (r = 0.149, p = 0.010) after controlling for field of study and year of study.

**Table 4:** Zero-order and partial correlations between knowledge, attitude, and practice scores

Variables	Zero-order correlation		Partial correlation^†^^†^	
	Coefficient (r)	*p* value	Coefficient (r)	*p* value
Knowledge-attitude	0.208	< 0.001	0.193	0.001
Knowledge-practice	0.353	< 0.001	0.343	< 0.001
Attitude-practice	0.159	0.006	0.149	0.010

^††^Controlled for field of study (0: Science, 1: Nonscience) and year of study (0: Years 1 – 2, 1: Years 3—4)

## Discussion

All universities were first closed in Malaysia when the Movement Control Order was implemented in response to the COVID-19 outbreak in March 2020. Since then, repeated university closures and movement restrictions have been implemented to combat the rise of COVID-19 infection. The knowledge, attitudes, and practices of students are important determinants in preventing and controlling the spread of the disease in the university environment.

The total percentage scores for the knowledge, attitude, and practice segments were 67.5%, 92.8%, and 72.4%, respectively. It was found the knowledge and practice scores of the current study were lower than the studies by Peng et al. [12], with 82% knowledge score and 89% attitude score for undergraduates in China, and Saefi et al. [17], with 74% knowledge score and 87% attitude score for undergraduates in Indonesia. Meanwhile, the attitude score obtained in the present study was greater than that of undergraduates in China (85%) and Indonesia (91%) [12, 17]. Based on Bloom’s cut-off (Good score: 80–100%, moderate score: 60–79%, and poor score: 0–59%) classification [18], the respondents in this study had good attitudes but moderate knowledge and practice levels towards COVID-19. As the adoption of COVID-19 preventative measures is strongly influenced by one’s knowledge [19], the government, health, and university authorities need to tailor a standardized nationwide COVID-19 education program to university students before going all out on reimplementing face-to-face teaching and learning.

Previous studies demonstrated that the KAP levels related to COVID-19 were affected by sex and ethnicity [17, 20]. However, the present study shows otherwise. This is attributable to the fact that Malaysia was once recorded as one of the highest daily COVID-19 cases in Southeast Asia in May 2021 [21]. The surge of COVID-19 cases at an alarming rate might prompt undergraduates, irrespective of sex and ethnicity, to seek information about the disease from different sources. Of these, social media were the main channel [22, 23]. Social media are crucial communication tools for information creation, dissemination, and consumption for the young generation. University students are one of the subset groups of the young generation [24]. The age range of the studied respondents was 18 to 25 years old (Table 1). Compared to other age groups, young people aged 16 to 24 years spend most of the time, approximately 3 h per day, on social media [25]. It is important to note that the information circulated on social media might not be entirely true. This is because the information posted by users on social media could be subjective and might contain conspiracy theories and misinformation [26]. Local and international health authorities, such as the Ministry of Health Malaysia, WHO, and Centers for Disease Control and Prevention (CDC), utilized their official accounts on various social media platforms to disseminate information about COVID-19. This could be a feasible way to promote healthy behavioral change to enhance COVID-19 preventative measures among students. However, research has found that only a number of university students obtained COVID-19-related information from official social media of health authorities [27]. This may explain the moderate knowledge and practice levels of the studied respondents.

Interestingly, our study demonstrated that the KAP scores of year 4 respondents were significantly lower (*p* < 0.05) than those of year 1 respondents. A study conducted among medical students also found that the KAP was influenced by year of study, but in a different trend, in which the KAP scores of year 4 students were greater than those of year 1 students [28]. The discrepancy might be due to the educational background of the respondents. Medical students generally begin their clinical training in year 4. They gain knowledge and understanding of different diseases during preclinical training. Hence, this group exhibits higher awareness of preventative measures for COVID-19 [29]. Additionally, many medical students involved themselves in managing COVID-19 patients during the pandemic [30]. Hence, it is not surprising that the KAP scores of year 4 medical students are greater than those of year 1 or preclinical students. In the present study, low KAP scores among year 4 students might be due to the large amount of time allocated to their final year project. Final year projects require students to work over a long period on data collection and analysis, background reading, and dissertation write-up [31]. This may result in their COVID-19 KAP scores being lowest compared to the respondents in academic years 1 to 3. Our current results suggest that COVID-19 workshops, seminars, or training programs focusing on year 4 students could be conducted, as a previous study indicated that the KAP of students improved after attending training related to COVID-19 [32].

Consistent with the findings of Hatabu et al. [24], respondents with science majors have a significantly greater (*p* < 0.05) attitude level towards COVID-19 than those without. Irrespective of the study majors, no difference in the COVID-19 knowledge and practice levels was detected. This might be attributed to the respondents having more time to online surf COVID-19-related information when studying at home or hostel [33], which indirectly enhances their level of knowledge.

Our findings also demonstrated positive correlations between knowledge-attitude, knowledge-practice, and attitude-practice, which were consistent with the literature data [17, 29]. Many studies have reported the importance of KAP in society to reduce the spreading rate of infection during pandemics and epidemics [24, 34]. This is because the attitudes of an individual towards an infection is related to the knowledge level of that infection, and this will substantially influence the practices or preventative measures targeted at mitigating the infection. However, knowledge alone may not account for attitudes and practices towards COVID-19, as other factors, such as emotional state and risk perception, impact respondents’ behavior and preventative measures [35]. This explains the weak interrelationship (r < 0.39) of the KAP in the present study.

This is possibly the first study assessing the KAP toward COVID-19 among Malaysian undergraduates during emergency remote learning. Insights generated from this study are useful for institutions and policymakers to formulate effective strategies to enhance the KAP of virtual learning undergraduates towards COVID-19 before reverting teaching and learning to the physical mode. Meanwhile, the use of convenience sampling hinders the representativeness of the data at the study population level.

## Conclusion

Teaching and learning in universities are conducted virtually during the COVID-19 outbreak. Accessing the KAP of COVID-19 among undergraduates is important before teaching and learning are reverted to the physical mode. Our study demonstrated that the total percentage scores of knowledge, attitude, and practice regarding COVID-19 among undergraduates were 67.5%, 92.8%, and 72.4%, respectively. This indicated that the respondents had good attitudes but moderate knowledge and practice levels toward COVID-19. Interventions focusing on raising the COVID-19 knowledge and practice levels of undergraduates are highly warranted.

## Data Availability

The data that support the findings of this study are available upon email request from the corresponding author.
